# Attenuation of the BTLA/HVEM Regulatory Network in the Circulation in Primary Sjögren’s Syndrome

**DOI:** 10.3390/jcm11030535

**Published:** 2022-01-21

**Authors:** Annabelle Small, Suzanne Cole, Jing J. Wang, Sunil Nagpal, Ling-Yang Hao, Mihir D. Wechalekar

**Affiliations:** 1 College of Medicine and Public Health, Flinders University, Adelaide, SA 5042, Australia; annabelle.small@flinders.edu.au (A.S.); jingjing.wang@flinders.edu.au (J.J.W.); 2 Discovery Immunology, Janssen R&D, 1400 McKean Road, Spring House, PA 19477, USA; scole10@ITS.JNJ.com (S.C.); lhao6@ITS.JNJ.com (L.-Y.H.); 3 Department of Immunology, SA Pathology, Flinders Medical Centre, Adelaide, SA 5042, Australia; 4 Immunology Research Unit, GSK, 1250 S Collegeville Road, Collegeville, PA 19426, USA; sunil.x.nagpal@gsk.com; 5 Department of Rheumatology, Flinders Medical Centre, Adelaide, SA 5042, Australia

**Keywords:** Sjögren’s syndrome, BTLA, HVEM, co-stimulation

## Abstract

Primary Sjögren’s syndrome (SjS) is an inflammatory autoimmune disorder which targets the lacrimal and salivary glands, resulting in glandular dysfunction. Currently, the immune drivers of SjS remain poorly understood and peripheral biomarkers of disease are lacking. The present study therefore sought to investigate the immune cell constituents of the SjS peripheral blood, and to assess the role of the BTLA/HVEM/CD160 co-stimulatory network by characterizing expression within the periphery. Peripheral blood mononuclear cells (PBMCs) were isolated from whole blood of *n* = 10 patients with SjS and *n* = 10 age- and sex-matched healthy control donors. Cells were divided and stained with three panels of antibodies, allowing assessment of T, B, and myeloid cell subsets, and measurement of BTLA, HVEM, and CD160 surface expression by flow cytometry. We identified distinct alterations in proportions of peripheral T, B, and myeloid cell types in SjS compared with healthy controls. Expression of BTLA/CD160/HVEM and frequency of BTLA/CD160/HVEM-expressing cells were significantly altered in peripheral SjS lymphocytes. The proportion of T cells co-expressing BTLA/HVEM and CD160/HVEM were significantly reduced in SjS. We found decreased BTLA and HVEM levels on peripheral B and T cells of SjS patients, and decreased BTLA/HVEM and CD160/HVEM co-expression, demonstrating dysregulation of the BTLA/HVEM axis in the peripheral blood of SjS patients. These results indicate the potential of targeting the BTLA-HVEM axis for the treatment of SjS.

## 1. Introduction

Activation of T lymphocytes requires multiple signals: (1) antigen binding to the T cell receptor (TCR), and (2) additional interaction with co-signaling molecules. These can exert stimulatory or inhibitory signals in the T cell, and act in a tightly controlled, coordinated manner to maintain homeostasis [1]. B and T lymphocyte attenuator (BTLA; CD272) is a co-signaling molecule of the CD28 immunoglobulin superfamily [2]. Similar to the well-characterized co-inhibitory molecules programmed cell death protein 1 (PD-1) and cytotoxic T-lymphocyte-associated protein 4 (CTLA-4), BTLA signaling in T cells inhibits cell activation and cytokine production [1]. This signaling is elicited through interaction with its ligand, herpes virus entry mediator (HVEM; TNFRSF14; CD270). Along with BTLA, HVEM has the additional interaction partners CD160 and LIGHT (TNFSF14; CD258). Together, the HVEM/BTLA/CD160/LIGHT molecules form an intricate, bi-directional, regulatory network capable of producing either co-stimulatory or co-inhibitory signals [3]. This complex network is important for the maintenance of self-tolerance; in cis, BTLA and HVEM form a heterodimeric complex which prevents HVEM binding in trans, preventing NF-κB signaling, and maintaining T cell tolerance [1]. Additionally, BTLA-expressing dendritic cells have been demonstrated to be critical for the differentiation of regulatory T (Treg) cells [4].

Primary Sjögren’s syndrome (SjS) is a chronic, inflammatory autoimmune disease which most commonly affects the lacrimal and salivary glands. While the specific immune drivers of SjS remain poorly understood, a breakdown of self-tolerance is central to disease, and thus, there is an interest in the role of checkpoint molecules in contributing to SjS. Specifically, deletion of CTLA-4 in adult mice has been shown to result in an autoimmune condition resembling human SjS [5], and BTLA knockout mice have been reported to develop symptoms characteristic of SjS [6]. Yet, despite these findings, the specific role of BTLA and its associated signaling pathway in SjS remains unstudied.

Here, we sought to examine the BTLA/HVEM/CD160/LIGHT co-signaling network in SjS to elucidate the role of this pathway in disease pathogenesis, to identify peripheral blood biomarkers of disease, and to investigate the potential of targeting this network therapeutically. Using flow cytometry, we investigated the expression, co-expression, and frequency of BTLA/HVEM/CD160-expressing cells in the peripheral blood of patients with primary SjS.

## 2. Materials and Methods

### 2.1. Human Subjects

Whole blood samples were collected from 10 SjS patients (mean age 66 years (range 55–82)) with anti-Ro/La autoantibodies and 10 age- and gender-matched healthy control donors. SjS patients fulfilled at least four of the six American–European Consensus Group Criteria. No other autoimmune conditions occurred within the study group, and none of the patients were being treated with any immunomodulatory medications at the time of blood sampling. Patient demographics are summarised in Table 1. The study was approved by the Southern Adelaide Human Research Ethics Committee, approval number 39.034.

### 2.2. Purification of Peripheral Blood Mononuclear Cells

Peripheral blood mononuclear cells (PBMCs) were isolated from whole blood samples using Lymphoprep™ (StemCell Technologies, Vancouver, Canada). Briefly, blood was diluted 1:1 in phosphate buffered saline (PBS) prior to being layered onto Lymphoprep™ and centrifuged for 20 min at 500× *g* with no brake. Following centrifugation, the PBMC-containing band was aspirated and washed in PBS (300× *g*, 10 min). Cells were counted using a haemocytometer and viability judged by their ability to exclude trypan blue. For cryopreservation, cells were cryopreserved in freezing media containing 10% foetal calf serum, 80% RPMI-1640 media, and 10% DMSO. Cells were incubated in a ‘Mr. Frosty’ Freezing Container (ThermoFisher Scientific, Waltham, MA, USA) overnight at −80 °C before transferal into liquid nitrogen for long-term storage. Prior to use, cryopreserved PBMCs were removed from storage and thawed rapidly at 37 °C before washing in RPMI-1640.

### 2.3. Flow Cytometry 

Thawed PBMCs were washed in 1 × PBS + 10% FCS. Cells were resuspended in PBS + 1% BSA at 1 × 10^6^ cells per test, and Fc receptors blocked using Human BD Fc Block™ (BD Biosciences). Following blocking, cells were stained with either myeloid, T cell, or B cell panels (summarised in Table S1). Following incubation, cells were washed in PBS + 1% BSA + 2 mM EDTA, resuspended in 4% paraformaldehyde, and incubated for 10 min at 4 °C in the dark. Cells were washed in PBS + 1% BSA + 2 mM EDTA prior to acquisition on a BD™ LSR II flow cytometer.

### 2.4. Statistical Analysis

To compare cell population frequencies and protein expression, GraphPad Prism 8.0 software (GraphPad, San Diego, CA, USA) was used to conduct two-tailed, unpaired Student’s *t*-test. Statistical significance was defined as *p* < 0.05.

## 3. Results

### 3.1. Altered PBMC Population Frequencies in SjS

**Figure 1:**
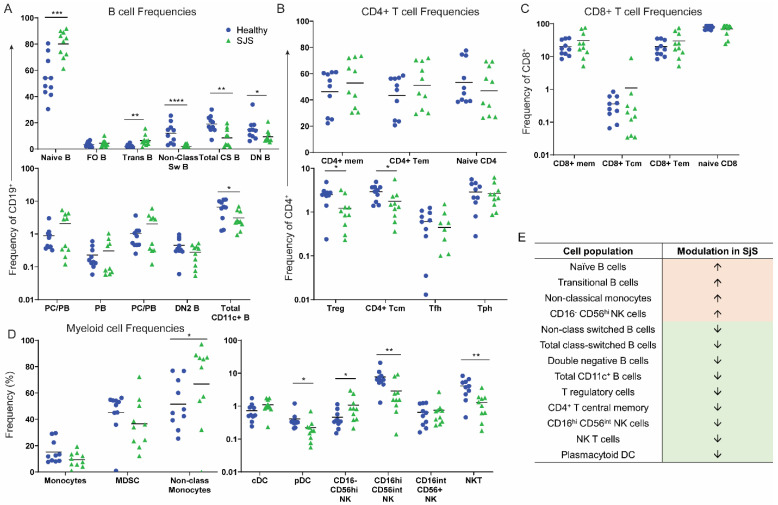
Peripheral blood mononuclear cell frequencies from patients with primary SjS (green) versus healthy controls (blue). (**A**) Frequencies of B cell populations, (**B**) CD4^+^ T cell populations, (**C**) CD8^+^ T cell populations, and (**D**) myeloid cell populations in SjS compared with healthy controls. (**E**) Summary of differences in cell frequencies in SjS. Data are expressed as geometric mean fluorescence intensity (gMFI) and are representative of *n* = 10 experiments, * *p* < 0.05, ** *p* < 0.01, *** *p* < 0.001, **** *p* < 0.0001, unpaired, two-tailed Student’s *t* test.

Firstly, we assessed for differences in immune cell frequencies in the blood between patients with SjS and healthy controls. We observed significantly increased proportions of naive B cells and transitional B cells, and decreased non-class switched B cells, class-switched B cells, double-negative B cells, and total CD11c^+^ B cells in SjS (Figure S1) (Figure 1A). In the T cell compartment (Figure S2), we observed decreased frequencies of T regulatory cells (Treg), CD4^+^ T effector memory (Tem) cells, CD8^+^ memory T cells, CD8^+^ T central memory (Tcm) cells, CD8^+^ Tem cells, and naïve CD8^+^ T cells compared with healthy controls (Figure 1B,C). In the myeloid compartment (Figure S3), we found increased frequencies of non-classical monocytes, and decreased frequencies of plasmacytoid dendritic cells, CD16^−^CD56^hi^ NK cells, CD16^hi^CD56^int^ NK cells, and NKT cells (Figure 1D). Summary of differences in cell frequencies is shown in Figure 1E.

### 3.2. BTLA/HVEM/CD160 Expression in SjS

**Figure 2:**
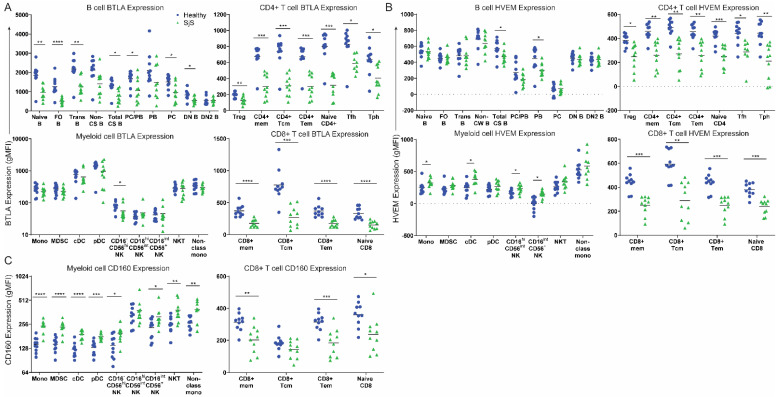
Expression of the BTLA/HVEM/CD160 regulatory network in T, B and myeloid compartments from SjS peripheral blood (green) compared with healthy controls (blue). Surface expression of (**A**) BTLA, (**B**) HVEM, and (**C**) CD160 in the indicated cell populations are shown. Data are expressed as geometric mean fluorescence intensity (gMFI) and are representative of *n* = 10 experiments, * *p* < 0.05, ** *p* < 0.01, *** *p* < 0.001, **** *p* < 0.0001, unpaired, two-tailed Student’s *t* test.

Next, we examined cell surface expression of BTLA, HVEM, and CD160. In SjS, we observed significantly reduced expression of BTLA in most B cells, and all assessed T cell populations, while we found no difference between myeloid populations (Figure 2A). These were reflected by reduced frequencies of BTLA-expressing B cells and most BTLA-expressing T cells (Figure S4).

HVEM expression was largely unchanged in SjS B cells compared with healthy cells, aside from a significant reduction in class-switched B cells and plasmablasts. Similarly to BTLA, we found reduced HVEM expression on all assessed T cell populations (Figure 2B), reflected by reduced frequencies of HVEM^+^ T cells (Figure S4B). Conversely, in myeloid cells, we found increased HVEM expression on classical monocytes (mono), conventional dendritic cells (cDC), CD16^hi^CD56^int^ NK cells, and CD16^int^CD56^+^ NK cells (Figure 2B).

CD160, the additional binding partner of HVEM, is expressed by CD8^+^ T cells and myeloid subsets. Thus, we next investigated CD160 expression on these cells in SjS. In CD8^+^ T cells, we found reduced CD160 expression on CD8^+^ memory T cells, CD8^+^ Tem, and naïve CD8^+^ T cells (Figure 2C). This was reflected by reduced frequencies of all CD160-expressing CD8^+^ populations (Figure S4). Conversely, in the myeloid compartment, we found significantly increased CD160 expression on all assessed populations, aside from CD16^hi^CD56^int^ NK cells (Figure 2C).

We additionally attempted to assess expression of the alternative HVEM binding partner LIGHT (TNFSF14) on SjS and healthy T cells but found this expression to be undetectable (not shown). This is likely due to the rapid and transient nature of LIGHT expression on activated T cells.

### 3.3. Checkpoint Molecule Co-Expression

**Figure 3:**
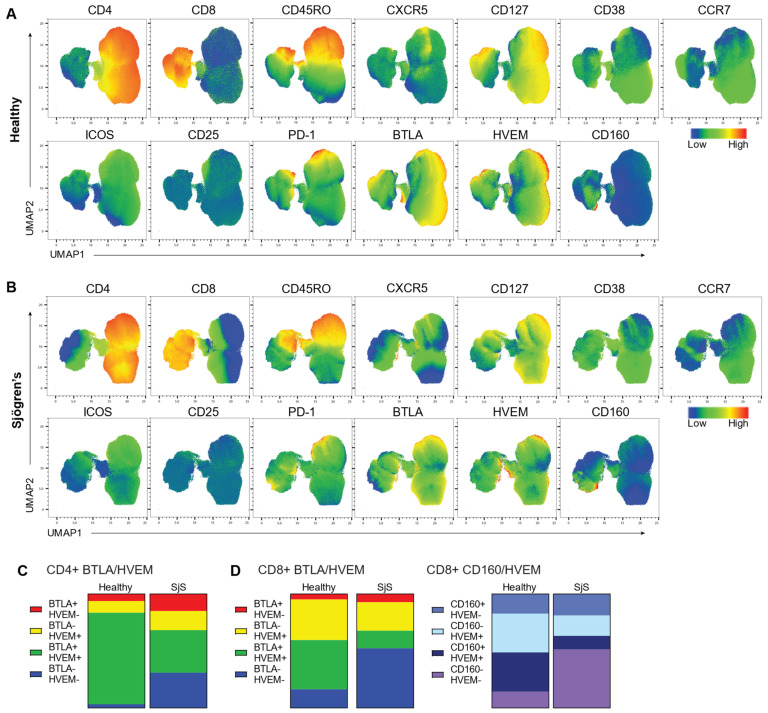
Checkpoint molecule co-expression in lymphocytes of SjS patients versus healthy controls. UMAP plots of flow cytometry data from (**A**) healthy control and (**B**) SjS lymphocytes. Color indicates cellular expression of labelled marker. (**C**) Summary of CD4^+^ lymphocyte BTLA/HVEM co-expression in healthy controls compared with SjS. (**D**) Summary of CD8^+^ lymphocyte BTLA/HVEM and CD160/HVEM co-expression in healthy controls compared with SjS. For (**C**,**D**), data are expressed as proportions of CD4^+^ or CD8^+^ T cells and are representative of *n* = 10 experiments.

In naïve T cells, BTLA and HVEM interact in cis to constitutively exert inhibitory signaling [3]. CD160 is similarly able to interact with HVEM in cis to suppress T cell function, or in a stimulatory manner on NK cells [7]. Thus, it was of interest to investigate BTLA/HVEM and CD160/HVEM co-expression in SjS lymphocytes. To do this, we utilized uniform manifold approximation and projection (UMAP) to visualize our data and compare populations between healthy controls (Figure 3A) and SjS (Figure 3B). In CD4^+^ cells (Figure 3C), we found that levels of co-expression of BTLA/HVEM were indeed altered in SjS. Proportions of BTLA^−^HVEM^−^ CD4^+^ cells were significantly enriched in SjS samples (*p* = 0.0021) along with BTLA^+^HVEM^−^ (*p* = 0.0039) and BTLA^−^HVEM^+^ cells (*p* = 0.0268), while BTLA^+^HVEM^+^ CD4^+^ cells were reduced (*p* < 0.0001). Similarly, in CD8^+^ T cells (Figure 3D), BTLA^−^HVEM^−^ cells were enriched (*p* < 0.0001), while BTLA^−^HVEM^+^ (*p* = 0.0083) and BTLA^+^HVEM^+^ (*p* < 0.0001) cells were reduced. BTLA^+^HVEM^−^ cells were unchanged. Meanwhile, CD160^−^HVEM^−^ cells were enriched (*p* < 0.0001), CD160^−^HVEM^+^ (0.001) and CD160^+^HVEM^+^ cells (*p* < 0.0001) were reduced, and CD160^+^HVEM^−^ cells were unchanged (Figure 3D).

## 4. Discussion

The high impact of SjS on patient quality of life and the large economic burden current symptomatic treatments impose suggest a need for new, improved therapeutic strategies [8]. This investigation has identified distinct expression changes in the BTLA/CD160/HVEM regulatory network in SjS and suggests the potential of targeting this pathway as a future therapeutic strategy.

We have identified distinct differences between the SjS and healthy peripheral blood. In SjS, we observed decreased proportions of CD27^+^ class-switched and non-class switched B cells, double negative B cells, and CD11c^+^ B cells, supporting previous findings [9]. This was reflected by increased naïve B cells and transitional B cells, supporting impaired B cell homeostasis in SjS [10]. Outside of the B cell compartment, the immune cell profile of our SjS cohort was characterized by decreased frequencies of Treg, Tcm, pDC, CD16^−^CD56^int^ NK and CD16^hi^CD56^int^ NK cells, and NKT cells, and an increased frequency of non-classical monocytes.

On SjS B cells, there was minimal modulation in HVEM expression compared with healthy controls, in contrast with significant decreases in BTLA expression on almost all assessed populations. Ligation of BTLA with HVEM in cis on B cells mediates B cell function similarly to T cells through inhibiting cell proliferation, cytokine production, and the up-regulation of other co-stimulatory molecules [11]. Thus, our finding of reduced BTLA expression on SjS B cells suggests that there is a lack of immune control in the form of dysregulated BTLA-mediated inhibitory regulation in SjS B cells.

In both CD4^+^ and CD8^+^ T cells, we found significantly reduced expression of BTLA (Figure 2A), and this was reflected by reductions in BTLA-expressing cell frequencies (Figure S4). Initial characterization studies of BTLA on healthy human cells report a steady reduction in BTLA expression upon activation of CD4^+^ T cells [12]. While this was the case in our healthy controls, in our cohort of SjS PBMCs, we did not observe a difference in the expression levels of BTLA by naïve CD4^+^ T cells and CD4^+^ T_mem_ or in the BTLA^+^ cell frequencies. These findings therefore suggest that signaling provided to T cells through BTLA is decreased in SjS, and that the regulation of BTLA expression in CD4^+^ T cells upon activation is impaired.

Activation of HVEM expressed by T cells induces activation of NF-κB and promotes cell survival [3]. In our cohort, HVEM expression was reduced in all T cell populations, suggesting that T cell survival signaling through HVEM may be impaired. Engagement of HVEM on a T cell by BTLA^+^ DC can induce the differentiation of Tregs [4]. Thus, our finding of decreased Tregs in SjS PBMCs could be explained by the decreased expression of HVEM on T cells.

Reduced CD160 expression in CD8^+^ T cells has been associated with autoimmunity [13]. Here, we found reduced expression of CD160 and reduced frequencies of CD160-expressing CD8^+^ cells in SjS. However, on myeloid cells, we found increased CD160 expression, particularly by CD16^−^CD56^hi^ and CD16^int^CD56^+^ NK cells. On NK cells, CD160 is important for the production of interferon-γ (IFNγ) [14]. In SjS, an ‘IFN-signature’ whereby IFN-inducible genes are enriched in salivary glands and peripheral blood has been described [15]. Thus, increased CD160 on peripheral blood NK cells may be a contributing cell type to this signature.

In cis, BTLA/HVEM and CD160/HVEM interactions exert inhibitory signals to maintain tolerance, or T cell naivety. We found reduced BTLA/HVEM co-expression on CD4^+^ and CD8^+^ T cells, and reduced CD160/HVEM co-expression on CD8^+^ T cells in SjS. This suggests that the cis regulatory interactions between BTLA/HVEM and CD160/HVEM are imbalanced in SjS.

Disease activity (measured by ESSDAI) of our patient cohort ranged from 2–14, with four patients measuring low disease activity (<5), and six patients measuring moderate-high activity (≥5). Despite observing significant changes in immune cell frequencies and expression of the BTLA regulatory network members in the SjS peripheral blood, regression modelling between these parameters and ESSDAI scores did not reveal significant correlations. Although this is not infrequent in clinical practice where patient-reported severity as measured by the ESSPRI (EULAR Sjogren’s Syndrome Patient Reported Index) does not necessarily correlate with ESSDAI, it may also be the result of the spread of the ESSDAI measurements in our cohort.

In the present study, we characterized the expression of the BTLA/HVEM/CD160 regulatory network in the periphery of patients with SjS, identifying distinct differences compared with healthy controls. However, this study is not without limitations. We were unable to detect the alternative HVEM binding partner LIGHT on the surface of lymphocytes, or as a soluble form in the serum of patients with SjS and healthy controls. This is likely due to the rapid nature of LIGHT expression on T cells following an activating stimulus. The function of LIGHT–HVEM interactions are context specific; in trans, LIGHT binding to HVEM transduces a co-stimulatory response in the HVEM-expressing T cell while in cis, LIGHT expression negatively regulates that of HVEM [3]. Thus, LIGHT is a critical controller of the BTLA/HVEM/CD160 network, and future investigation into LIGHT and its role in SjS are needed to fully understand the role of this pathway in SjS and other autoimmune conditions.

## Figures and Tables

**Table 1:** Patient demographics.

Patient Characteristics (*n* = 10)	
Gender (% male/female)	20/80
Age (years)	Mean: 65.6 Min: 55 Max: 80
Glandular enlargement (% yes/no)	70/30
Serology (% pos/neg)	
Anti-Ro60	100/0
Anti-Ro52	100/0
Anti-La	70/30
EULAR Sjogren’s Syndrome disease activity index (ESSDAI)	Mean: 7 Min: 2 Max: 14

## Data Availability

The data supporting this study are available within the paper and Supplementary Information. Any additional data relating to the study are available from the corresponding author on reasonable request.

## Supplementary Materials

The following supporting information can be downloaded at: https://www.mdpi.com/article/10.3390/jcm11030535/s1. Table S1: Full list of antibodies used in this study for flow cytometric purposes, their conjugates, sources, catalog numbers, and clone numbers. Figure S1: B cell gating strategy; Figure S2: T cell gating strategy; Figure S3: Myeloid gating strategy; Figure S4: BTLA/HVEM/CD160-expressing cell frequencies in T, B, and Myeloid compartments from SjS peripheral blood compared with healthy controls.
