# Supplementary material for: Attenuation of the BTLA/HVEM Regulatory Network in the Circulation in Primary Sjögren’s Syndrome
Source: J Clin Med. 2022 Jan 21;11(3):535. doi: 10.3390/jcm11030535 (PMC8837091; doi:10.3390/jcm11030535)
Supplement: Supplementary file 1 [file jcm-11-00535-s001.zip › jcm-1559005-supplementary.pdf]

Supplementary Materials

Attenuation of the BTLA/HVEM Regulatory Network in the Circulation in Primary Sjögren's Syndrome

Annabelle Small [1], Suzanne Cole [2], Jing J. Wang [1,3], Sunil Nagpal [4], Ling-Yang Hao [2] and Mihir D. Wechalekar [1,5,*]

1 College of Medicine and Public Health, Flinders University, Adelaide, SA 5042, Australia;
annabelle.small@flinders.edu.au (A.S.); jingjing.wang@flinders.edu.au (J.J.W.)
2 Discovery Immunology, Janssen R&D, 1400 McKean Road, Spring House, PA 19477, USA; scole10@ITS.JNJ.com (S.C.); lhao6@ITS.JNJ.com (L.-Y.H.)
3 Department of Immunology, SA Pathology, Flinders Medical Centre, Adelaide, SA 5042, Australia
4 Immunology Research Unit, GSK, 1250 S Collegeville Road, Collegeville, PA 19426, USA; sunil.x.nagpal@gsk.com
5 Department of Rheumatology, Flinders Medical Centre, Adelaide, SA 5042, Australia
* Correspondence: mihir.wechalekar@sa.gov.au

Table S1. *Full list of antibodies used in this study for flow cytometric purposes, their conjugates, sources, catalog numbers, and clone numbers.*

Antigen	Fluorophore	Vendor	Catalog #	Clone
**Myeloid**				
**CD160**	AF488	Invitrogen	53-1609-42	BY55
**CD33**	PE	BioLegend	303404	WM53
**BTLA**	PE Dazzle	BioLegend	344522	MIH26
**HLA-DR**	PerCP-Cy5.5	BD Biosciences	552764	G46-6
**CD11b**	PE-Cy7	BD Biosciences	557743	ICRF44
**HVEM**	APC	BioLegend	318808	122
**CD3**	AF700	BioLegend	317340	OKT3
**L/D-NearIR**	APC-Cy7	Invitrogen	L10119	N/A
**BDCA2 (CD303)**	BV421	BioLegend	354212	201A
**CD16**	BV510	BD Biosciences	563830	3G8
**CD123**	BV605	BioLegend	306026	6H6
**CD56**	BV650	BioLegend	318344	HCD56
**CD11c**	BV711	BD Biosciences	563130	B-ly6
**CD14**	BV785	BD Biosciences	563698	M5E2
**T Cell**				
**CD160**	AF488	Invitrogen	53-1609-42	BY55
**PD1**	PE	Biolegend	32996	EH12.2H7
**BTLA**	PE Dazzle	Biolegend	344522	MIH26
**CD45RO**	PerCP-Cy5.5	BD Biosciences	560607	UCHL1
**CD25**	PECy7	Invitrogen	25-0259-42	BC96
**HVEM**	APC	Biolegend	318808	122
**CD8**	AF700	Biolegend	301028	RPA-T8
**L/D-NearIR**	APC-Cy7	Invitrogen	L10119	N/A
**CD56**		Biolegend	318332	HCD56
**CXCR5**	BV421	Biolegend	356920	J252D4
**CD4**	BV510	Biolegend	317444	OKT4
**ICOS**	BV605	Biolegend	313538	C398.4A
**CD38**	BV650	BD Biosciences	740574	HIT2
**CCR7**	BV711	BD Biosciences	563712	3D12
**CD127**	BV785	Biolegend	351330	A019D5
**B Cell**				
**CD160**	AF488	Invitrogen	53-1609-42	BY55
**CD138**	PE	Biolegend	356504	MI15
**BTLA**	PE-Dazzle	Biolegend	344522	MIH26
**CD24**	PerCP	BD Biosciences	Ml5	561647
**IgD**	PECy7	BD Biosciences	561314	IA6-2
**HVEM**	APC	Biolegend	318808	122
**CD20**	AF700	Biolegend	302322	2H7
**L/D-NearIR**	APC-Cy7	Invitrogen	L10119	N/A
**CD27**	BV421	Biolegend	356418	M-T271
**IgM**	BV510	BD Biosciences	563113	G20-127
**CD11c**	BV605	BD Biosciences	563929	B-ly6
**CD38**	BV650	BD Biosciences	740574	HIT2
**CD19**	BV711	Biolegend	363022	SJ25C1
**CD3/CD56**	BV785	Biolegend (CD3)<br>BD (CD56)	300472<br>564058	UCHT1<br>NCAM16.2

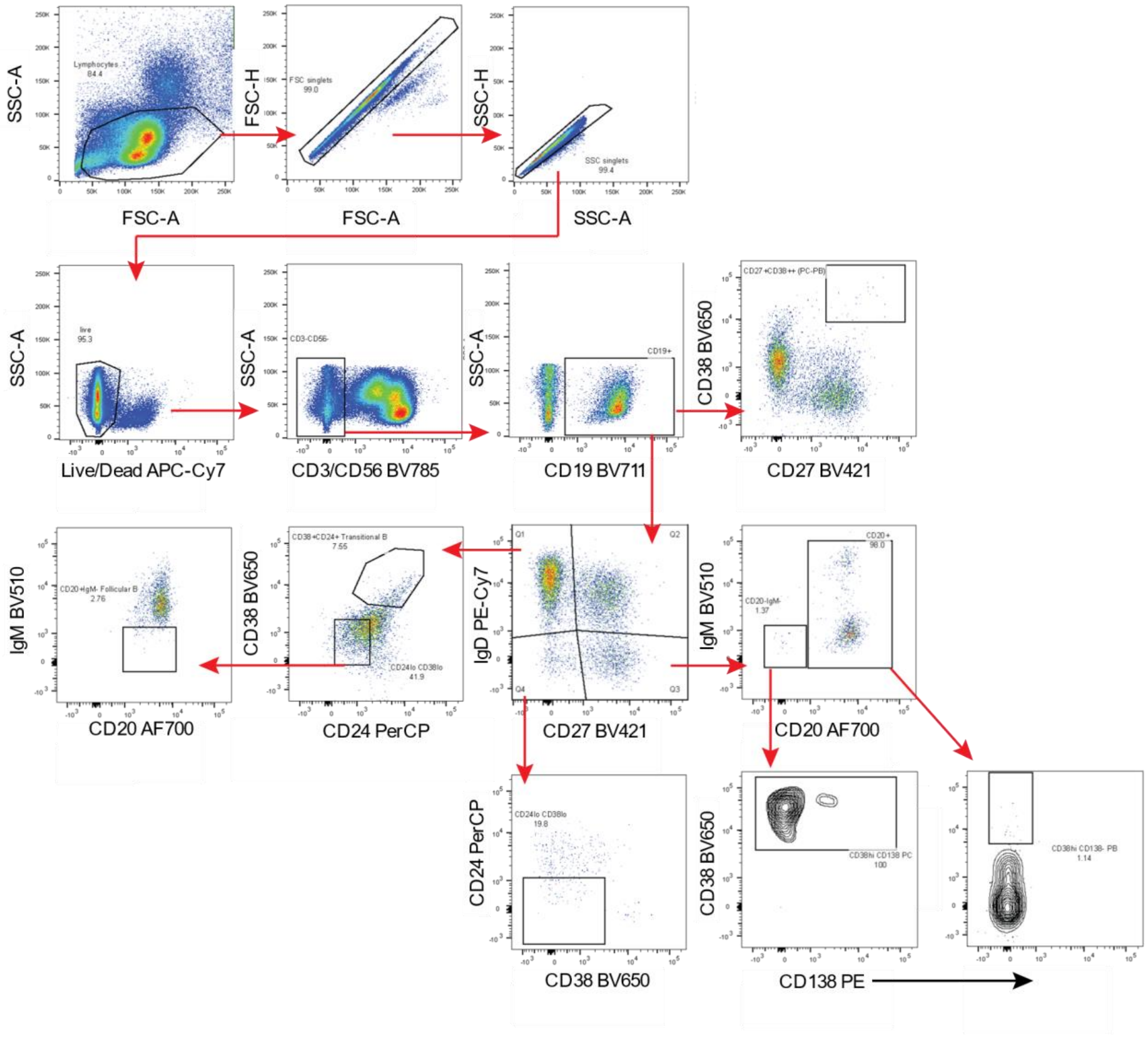

Figure S1. *B cell gating strategy*. Representative plots show B cell population gating by capture of lymphocytes by FSC/SSC, followed by doublet exclusion by FSC-H vs. FSC-A then SSC-H vs. SSC-A, and then exclusion of APC-Cy7^{+} cells (non-viable), and then exclusion of CD3/CD56^{+} cells. Gating of different B cell populations is then as shown.

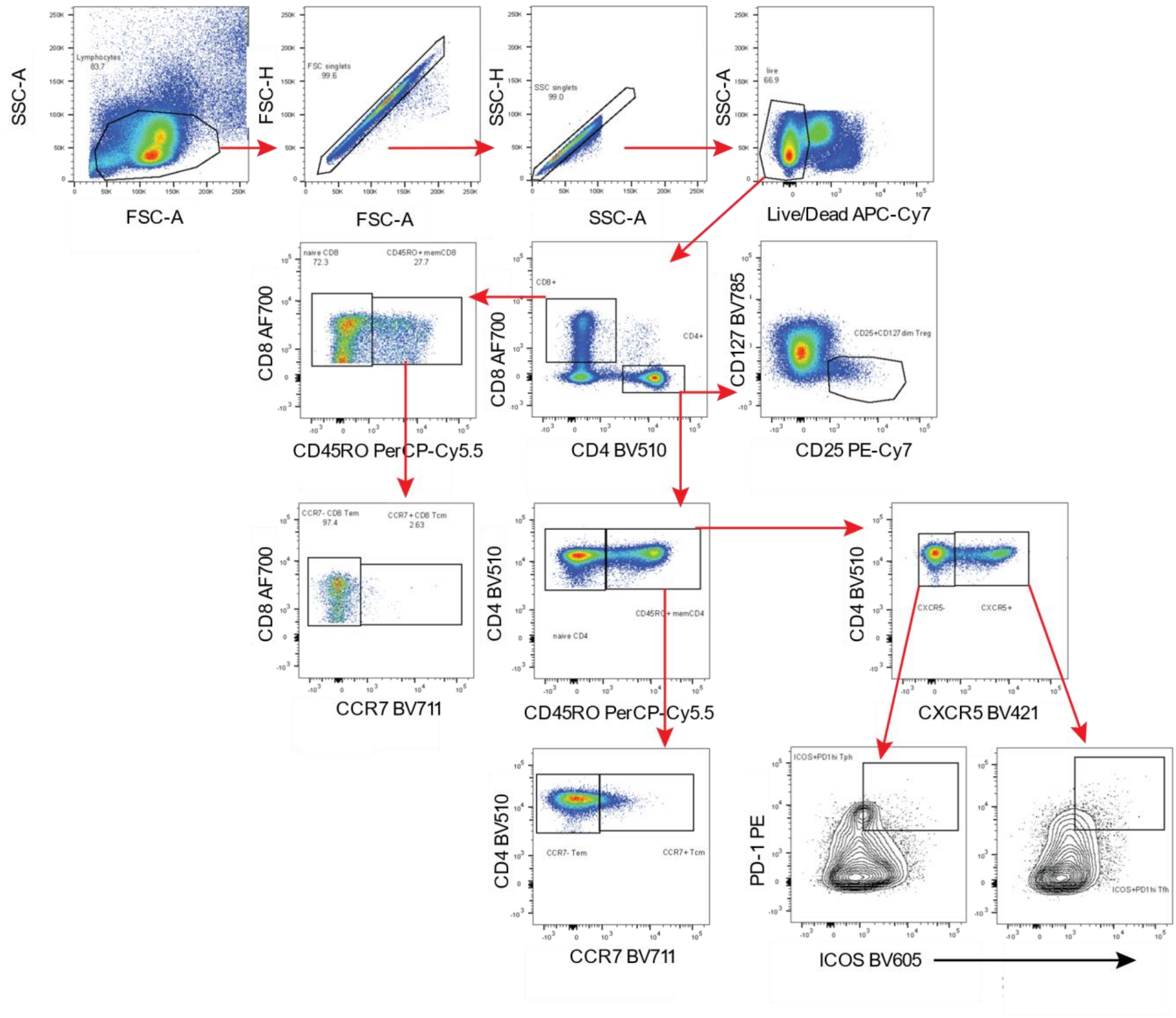

Figure S2. *T cell gating strategy.* Representative plots show T cell population gating by capture of lymphocytes by FSC/SSC, followed by doublet exclusion by FSC-H vs. FSC-A then SSC-H vs. SSC-A, and then exclusion of APC-Cy7^{+} cells (non-viable), and then subsequent gating of CD8^{+} and CD4^{+} and downstream populations as shown.

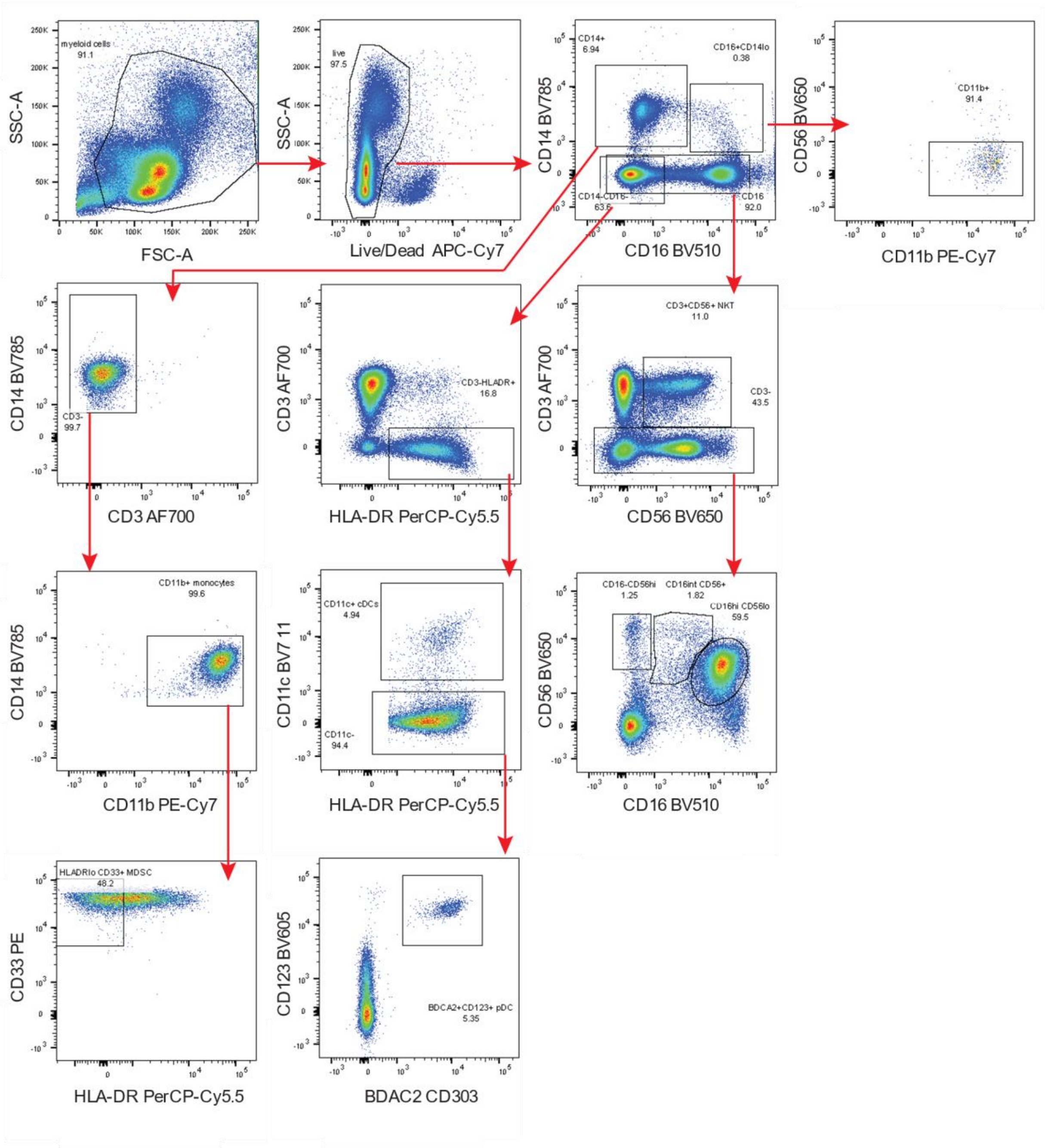

Figure S3. *Myeloid gating strategy*. Representative plots show myeloid population gating by capture of lymphocytes and larger cells by FSC/SSC, followed by exclusion of APC-Cy7^{+} cells (non-viable), and then subsequent gating of CD14$^{+/-}$ and CD16$^{+/-}$ populations as shown above.

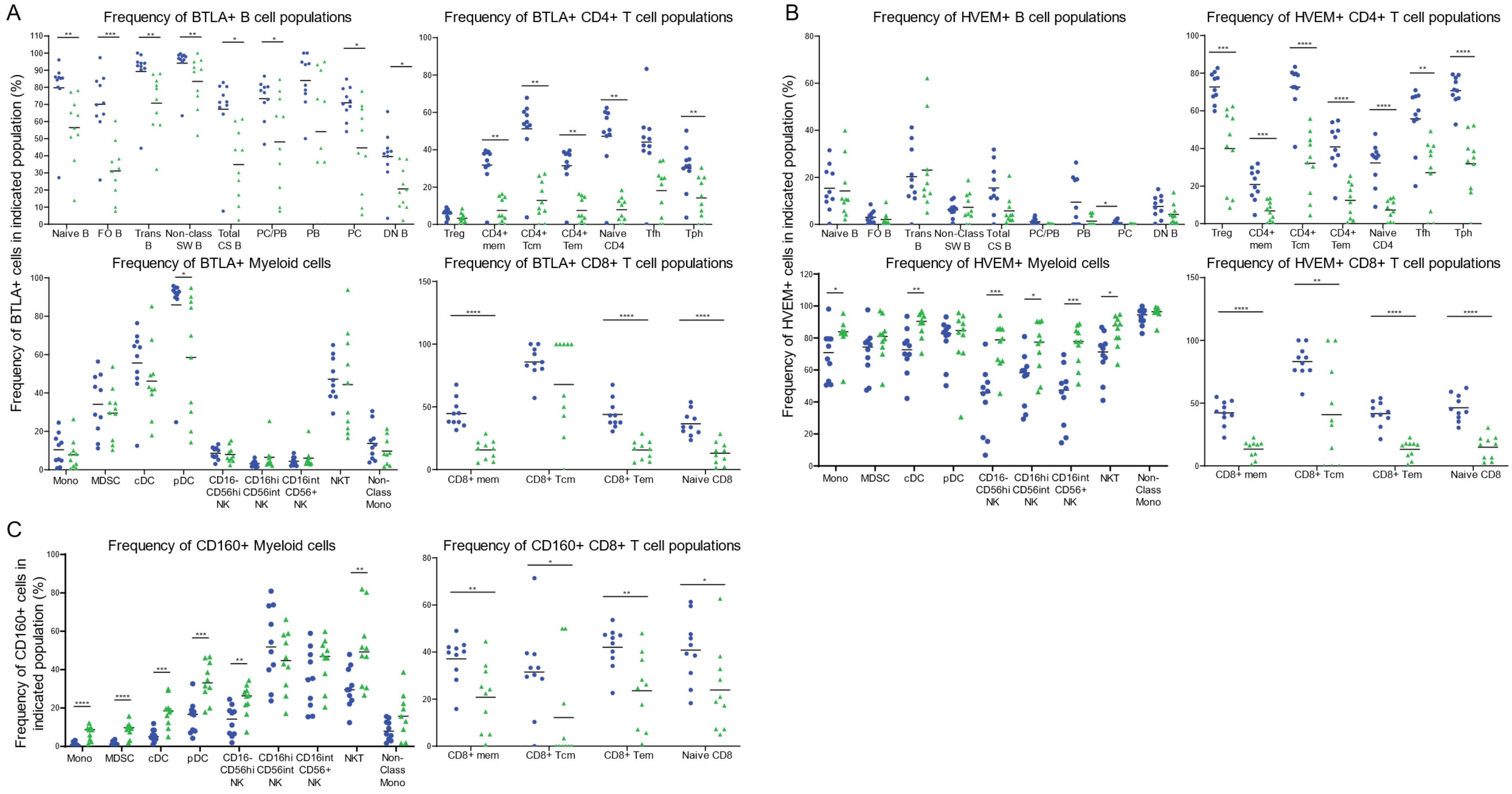

Figure S4. *BTLA/HVEM/CD160-expressing cell frequencies in T, B, and Myeloid compartments from SjS peripheral blood (green) compared with healthy controls (blue).* Frequency of (A) BTLA-, (B) HVEM-, and (C) CD160-expressing cells in the indicated cell populations are shown. Data is expressed as proportions of the indicated population (%), and is representative of n=10 experiments, *$p<0.05$, **$p<0.01$, ***$p<0.001$, ****$p<0.0001$, unpaired, two-tailed student's *t* test.
